# Frequency-modulated atomic force microscopy localises viscoelastic remodelling in the ageing sheep aorta

**DOI:** 10.1016/j.jmbbm.2016.07.018

**Published:** 2016-12

**Authors:** R. Akhtar, H.K. Graham, B. Derby, M.J Sherratt, A.W. Trafford, R.S. Chadwick, N. Gavara

**Affiliations:** a Centre for Materials and Structures, School of Engineering, University of Liverpool, L69 3GH UK; b Institute of Inflammation and Repair, Manchester Academic and Health Sciences Centre, Stopford Building, The University of Manchester, Oxford Road, Manchester, M13 9PT UK; c School of Materials, The University of Manchester, Oxford Road, Manchester, M13 9PL UK; d Institute of Cardiovascular Sciences, Faculty of Medical and Human Sciences, The University of Manchester, 46 Grafton Street, Manchester, M13 9NT UK; e Auditory Mechanics Section, National Institute on Deafness and Other Communication Disorders, National Institutes of Health, Bethesda, MD, USA; f School of Engineering and Materials Science, Queen Mary University of London, Mile End Road, London, E1 4NS UK

**Keywords:** Frequency-modulated atomic force microscopy, Aorta, Ageing, Lamellar, Medial layer

## Abstract

Age-related aortic stiffening is associated with cardiovascular diseases such as heart failure. The mechanical functions of the main structural components of the aorta, such as collagen and elastin, are determined in part by their organisation at the micrometer length scale. With age and disease both components undergo aberrant remodelling, hence, there is a need for accurate characterisation of the biomechanical properties at this length scale. In this study we used a frequency-modulated atomic force microscopy (FM-AFM) technique on a model of ageing in female sheep aorta (young: ~18 months, old: >8 years) to measure the micromechanical properties of the medial layer of the ascending aorta. The novelty of our FM-AFM method, operated at 30 kHz, is that it is non-contact and can be performed on a conventional AFM using the ׳cantilever tune’ mode, with a spatial (areal) resolution of around 1.6 μm^2^. We found significant changes in the elastic and viscoelastic properties within the medial lamellar unit (elastic lamellae and adjacent inter-lamellar space) with age. In particular, there was an increase in elastic modulus (Young; geometric mean (geometric SD)=42.9 (2.26) kPa, Old=113.9 (2.57) kPa, *P*<0.0001), *G*′ and *G*″ (storage and loss modulus respectively) (Young; *G*′=14.3 (2.26) kPa, Old *G*′=38.0 (2.57) kPa, *P*<0.0001; Young; *G*″=14.5 (2.56) kPa, Old *G*″=32.8 (2.52) kPa, *P*<0.0001). The trends observed in the elastic properties with FM-AFM matched those we have previously found using scanning acoustic microscopy (SAM). The utility of the FM-AFM method is that it does not require custom AFM hardware and can be used to simultaneously determine the elastic and viscoelastic behaviour of a biological sample.

## 1 Introduction

Ageing is associated with arterial stiffening and the development of cardiovascular disease. Stiffening in large arteries such as the aorta is typically studied at the level of the whole vessel. However, there is now evidence which suggests that alterations in the structure and mechanical properties of large arteries at the micron scale can have detrimental effects on the function of the aorta (Kohn et al., 2015). Hence, there is growing interest in characterizing the nano- and micro-mechanical properties of the aorta to better understand the structure-property-function relationships within large arteries (Akhtar et al., 2014, Akhtar et al., 2009, Grant and Twigg, 2013, Kohn et al., 2015). Due to the intricate organisation of extracellular matrix (ECM) components within the vessel wall, there is a need to develop methods that accurately measure the mechanical properties of the tissue with high spatial resolution. However, due to a lack of appropriate techniques, there is still limited information on these properties at the micron length scale (Graham et al., 2011). Accessing this information is vital because both force-sensing cells and large ECM assemblies such as collagen and elastic fibres, which govern the mechanical response of large arteries, are organised at this length scale (Akhtar et al., 2011). Hence, there is a need to develop reliable, quantitative methods for the assessment of the mechanical properties of vascular tissue at the micron length scale (Akhtar, 2014, Akhtar et al., 2009).

Our previous work focussed on the use of scanning acoustic microscopy (SAM) to determine regional variations in the elastic properties of the aorta at this length scale (Akhtar et al., 2014, Graham et al., 2011, Lopez-Andres et al., 2012). SAM measures the speed of longitudinal acoustic waves through a material, which can be related to its elastic modulus. When SAM is operated at frequencies close to 1 GHz, it provides quantitative measurements of acoustic wave speed with a linear spatial resolution around 1 μm. Using this technique we have shown that changes in the micromechanical properties of the aorta that occur with age and with pathology (diabetes), can be localised to the inter-lamellar regions of the medial lamellar unit (MLU) within the aorta. The MLU is the main load-bearing component of the aortic wall and is composed of an elastin-rich lamellae and a fibrillar collagen with a vascular smooth muscle cell-rich inter-lamellar region (O׳Connell et al., 2008).

Although the use of SAM for measuring the properties of soft tissues such as the aorta has a number of advantages, including co-localisation of histological and elastic properties and fast data acquisition (Zhao et al., 2012), SAM cannot provide information on the viscoelastic and time-dependant response of tissue, because it operates at low amplitude acoustic deformation rates, and is limited to characterising tissue stiffness indirectly because acoustic wave speed is a function of both elastic modulus and local density. Another technique for acquiring mechanical properties is a contact or indentation method such as atomic force microscopy (AFM) which can be used to measure the elastic and viscoelastic properties of tissues at a higher spatial resolution than SAM, and has been used, for example, to characterise intimal stiffening in the aorta (Huynh et al., 2011).

Despite the relatively wide use of AFM for biological applications, AFM-indentation approaches are not always ideal for probing soft biological tissues due to lengthy acquisition times and also the requirement of the probe to be either continuously or intermittently in contact with the sample. A comparison of acoustic and AFM methods for the characterisation of tissue has been reviewed elsewhere (Akhtar et al., 2011).

Frequency-modulated atomic force microscopy (FM-AFM) is an AFM mode which overcomes some of these issues and can be employed by existing commercial AFM systems. The principle of FM-AFM is that it relies on detecting small changes in the cantilever resonant frequency, which occur in response to the tip–sample interaction (Higgins et al., 2005). These frequency shifts can then be used to measure force interactions between the tip and sample. Although there are concerns about uncertainty when AFM techniques are used for mechanical property characterisation e.g. due to complications arising due to tip-sample interactions (Lin et al., 2009), AFM methods are nevertheless powerful tools for the nano- and micro-scale characterisation of soft tissues. When probing the difference between two samples, any uncertainty can be minimised by utilising the same tip and configuration to provide more robust mechanical property data.

FM-AFM is a powerful tool for studying the mechanical properties of single biomolecular interactions and force-extension response of isolated molecules in liquid (Higgins et al., 2005, Humphris et al., 2000). FM-AFM has previously been applied to biological systems to study single polysaccharide molecules (Humphris et al., 2000) and DNA (Cerreta et al., 2013), for example. Gavara and Chadwick (2010) developed the technique further, specifically for applications in the study of the microrheology of biological tissues that produce or detect sound. In that work, they demonstrated that material properties of gels and soft biological tissue can be determined via frequency modulation based on hydrodynamics theory of thin gaps that they developed. The key advantages of this method are that it overcomes the limitations of contact and tapping-mode AFM because the probe does not make any direct contact with the sample (Gavara and Chadwick, 2010). Further, the amount of force applied onto the sample is reduced to the scale of 10^−12^ N (piconewtons, pN). The technique was validated by characterising polyacrylamide gels of different stiffness, as well as determining regional variations in the elastic and viscoelastic properties of soft biological tissue in near-physiological conditions.

In this paper, we apply the FM-AFM method to examine the elastic and viscoelastic properties of young and old sheep aorta. We show that age-related changes in the viscoelastic properties of the tissue are localised to the inter-lamellar regions of the aortic medial layer.

## 2 Material and methods

### 2.1 Tissue samples

Testing was conducted on ascending aortae from young (~1.5 years, *n*=5) and old (>8 years, *n*=5) female sheep (Ovis aries). Sheep were killed by intravenous injection of pentobarbitone (200 mg/kg) (Dibb et al., 2004) and the ascending aorta rapidly removed,. The aorta was then frozen in optimal cutting temperature (OCT) compound (Sakura Finetek Europe B.V., The Netherlands) and transverse cryosections were subsequently taken with 5 µm thickness and adsorbed onto glass slides. The sections were kept frozen at −80 °C before being thawed and hydrated in Phosphate Buffered Saline (PBS) for mechanical testing.

### 2.2 Frequency-modulated atomic force microscopy

Measurements were performed using a Bioscope Catalyst AFM instrument (Bruker, Billerica, MA, USA) mounted on the stage of an Axiovert 200 inverted microscope (Carl Zeiss, Göttingen, Germany) placed on a vibration-isolation table (Newport Isostation, Irvine, CA, USA). A 10 μm diameter latex bead glued to a tipless V-shaped gold-coated silicon nitride cantilever (Veeco NP-020, Bruker, Billerica, MA, USA) was used as a probe. The spring constant of the cantilever was 0.1942 N m^−1^. As described in detail by Gavara and Chadwick (2010), FM-AFM was carried out using the ׳cantilever tune׳ feature of the tapping mode to sweep the cantilever drive frequency over a selectable frequency range. A key feature of the cantilever tune mode is the ability to perform frequency sweeps at a controlled height over the sample׳s surface with nanometre resolution (in the *z*-direction). Extensive details of the methodology and underlying theory are reported elsewhere (Gavara and Chadwick, 2010).

All measurements were performed with the tissue sections hydrated in PBS solution at room temperature. For each test, the cantilever tune mode was utilised to position the tip of the cantilever 200 nm above the sample. An initial frequency sweep was performed to locate *f*_π/2_ of interest, that is, the frequency at which we observed a *π*/2 phase difference between the oscillation of the piezo and the cantilever. In addition, the drive amplitude of the piezo was adjusted to ensure that the amplitude of oscillation of the cantilever at *f*_π/2_ was below 5 nm. Frequency sweeps were recorded with a 3 kHz frequency range around *f*_π/2_. Sweeps were performed for two distances between the bead and the sample, 50 nm and 200 nm. For each bead-sample distance, five sweeps were recorded in order to reduce inherent variability associated with AFM measurements. Thus, 10 frequency sweeps were used to calculate the mechanical properties for a given location i.e. each individual measurement was determined from 2 tip-sample distances, which were in turn averaged from 5 measurements at each of these distances (as outlined below in Section 2.3).

**Fig. 1:**
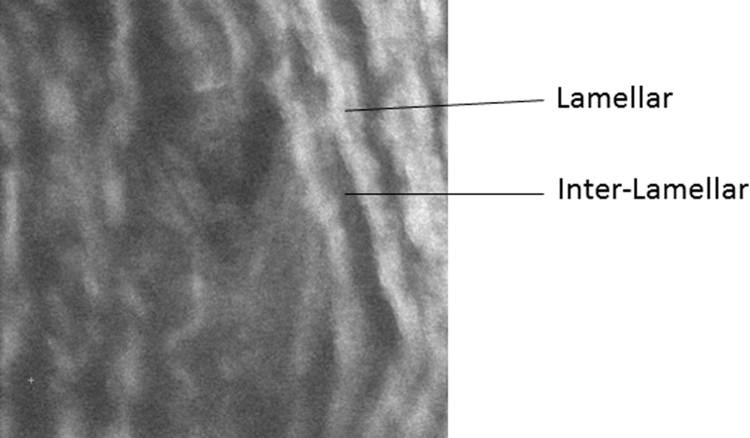
Example optical image captured with the integrated microscope for localisation of lamellar and inter-lamellar regions of the medial layer. The size of the image 150×150 µm obtained with a 40× objective (880×880 pixels).

To determine the overall properties of the medial layer of the aortic wall, 11 random locations were selected on 3 tissue sections for each animal. In addition, the fluorescence microscope incorporated within the AFM system was used to identify and locate lamellar and inter-lamellar regions of the tissue using elastin auto-fluorescence (Fig. 1) and 5 lamellar and 5 inter-lamellar locations were selected on 3 tissue sections for each animal.

### 2.3 Data processing

All data were processed using Matlab (Mathworks, Natick, MA, USA). For each recorded frequency sweep, phase-frequency curves were fitted with a second-order polynomial around the π/2 to determine *f*_*π*/2_. Values of *f*_*π*/2_ for the five frequency sweeps recorded at each height were used to determine a mean value. The theory to estimate elastic modulus (*E*) and effective viscosity (*µ_eff_*), using the changes in frequency and phase of cantilever oscillation has been developed elsewhere (Gavara and Chadwick, 2010). The formulas used are:

(1) $$E={\left(\frac{9k∆f}{c_{0}}\right)}^{3/2}\rho^{-1/2}a_{0}^{-2}{\left(2\pi f_{\infty }\right)}^{-5/2}{\left(1-\frac{6}{f}\frac{\mathrm{df}}{d\phi }\right)}^{-1}{\left(h_{200}^{1/3}-h_{50}^{1/3}\right)}^{-3/2}$$

(2) $$\mu_{\mathrm{eff}}=\frac{3E\frac{\mathrm{df}}{d\phi }}{\pi f^{2}}$$

where *c*_0_=0.3419; *k* is the stiffness of the cantilever, Δ*f* is the frequency shift (Hz) where the phase is π*/2*; *ρ* is the density of water, *a*_0_ is the radius of the bead attached to the end of the cantilever, *f* is the frequency where the phase is *π/2*; *f*_*∞*_ is the frequency far from the sample; *dϕ*/*df* is the slope of the phase-frequency curve where the phase is *π/2.*

We can then obtain further viscoelastic parameters such as storage shear modulus (*G*′), loss modulus (*G*″) and loss tangent (*G*″/*G*′) as follows:

(3) $$G^{\prime }=\frac{E}{2\left(1+\nu \right)}$$

(4) $$G^{\prime \prime }=2\pi f\mu_{\mathrm{eff}}$$

where *ν* is the Poisson׳s ratio of the sample and it is assumed to be 0.5.

The frequency shift used in these calculations was the mean value across 3 different tissue sections for each animal.

### 2.4 Statistical analysis

We carried out normality tests on the data distributions obtained, as well as on the logarithm of the data distributions. The results of the normality test indicate that the majority of the data obtained followed a log-normal distribution. Accordingly, results are expressed as geometric mean and geometric standard deviation, geomean (geoSD). Statistical analysis has been conducted with the non-parametric Mann-Whitney and Kruskal-Wallis ANOVA tests.

## 3 Results

### 3.1 Mechanical remodelling in the medial layer

**Fig. 2:**
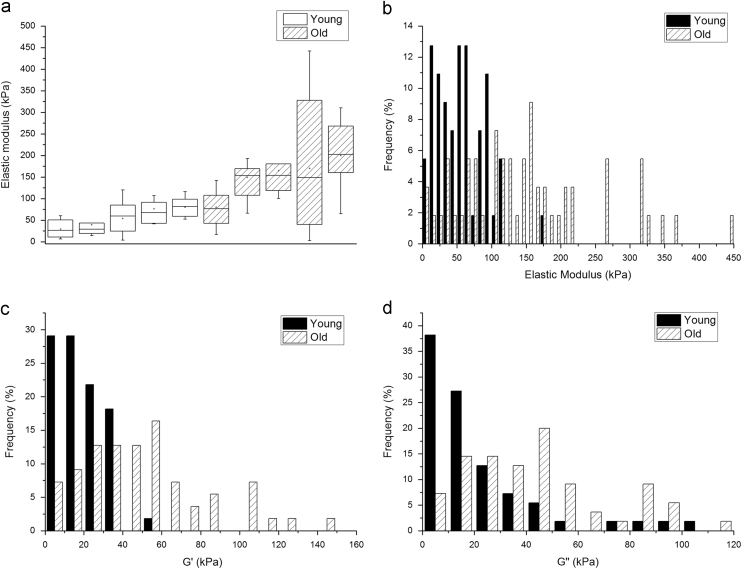
a) Individual and inter-animal variations in the mechanical properties of the medial layer. a) Elastic modulus of the medial layer for each animal in the young and old group b) Frequency distribution of the elastic modulus c) Frequency distribution of *G*′ and d) Frequency distribution of *G*″ in the young and old sheep aorta groups (*n*=55 measurements/group with 11 measurements/animal).

The variation in mechanical properties between individual animals is shown in Fig. 2a. With the exception of one individual sample, the mean and median values were higher in the old group as compared to the young group. All of the remaining data are presented comparing overall trends in the young vs old group. *E*, *G*′ and *G*″ were all significantly higher in the old group as compared with the young group (Table 1). The frequency distributions (young vs old) were relatively similar for *E*, *G*′ and *G*″ (Fig. 2b–d).

The loss tangent was lower in the old group as compared to the young group (young=1.0 (1.94); old=0.86 (1.2)) but the difference was not significant (*P*=0.28). Finally, when comparing the intra-tissue versus inter-animal variability of our data, we found that the CoV (coefficient of variance) was largest for data obtained at different locations of the same tissue, rather than the CoV arising from data from different animals (intra-tissue CoV=26.5±16.7 (mean±SD); inter-animal CoV=16.6±7.7 (mean±SD) but the difference was not significant (*P*=0.09).

### 3.2 Localised mechanical properties of lamellar and inter-lamellar regions

**Fig. 3:**
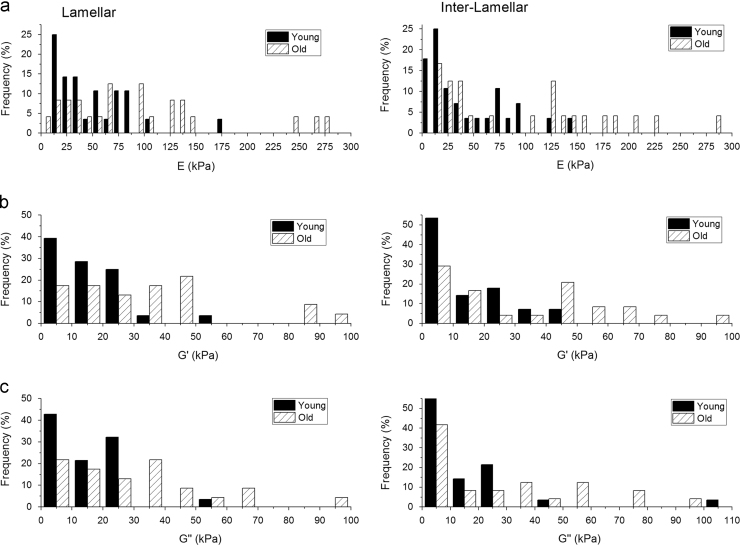
Frequency distribution for the lamellar and inter-lamellar regions of the medial layer for a) Elastic modulus b) *G*′ c) *G*″ in the young and old sheep aorta groups (*n*=24 measurements/group). The histogram distributions were found to be statistically significant in each group (Kruskal-Wallis ANOVA, *P*<0.05 for lamellar regions and *P*<0.01).

The elastic modulus frequency distributions were profoundly different in the lamellar and inter-lamellar regions of the media, as shown in Fig. 3. The mean data are summarised in Table 2. The elastic modulus was significantly higher in the old group for both the lamellar and inter-lamellar regions of the medial layer. Both *G*′ and *G*″ increased with age in the lamellar and inter-lamellar regions.

## 4 Discussion

The FM-AFM technique is shown to be a powerful tool for quantitatively assessing regional differences in the mechanical properties of soft tissues such as the aorta. As applied in this study, it has provided new insights into the age-related viscoelastic behaviour of the ovine aorta. This is the first study, to the best of our knowledge, to show how both the elastic and viscoelastic properties vary within the medial lamellar unit with age. FM-AFM overcomes the various issues of contact or intermittent contact mode AFM when used for mechanical measurements, such as using the appropriate contact mechanics model for accounting for tip-sample interaction (Lin et al., 2009). The main advantage of this technique is that it is a non-contact method, which means that much lower forces and hence local deformations are applied to soft, biological tissues than with other AFM-based indentation techniques (Gavara and Chadwick, 2010). Given that experiments in this study were conducted on thin cryosections of tissue, it seems that the low force applied with this non-contact avoids the overestimation of the mechanical properties of the tissue due to the glass substrate, which is a typical challenge with AFM and nanoindentation techniques (Akhtar et al., 2011).

Additionally, a significant advantage of the FM-AFM method is that no hardware modifications are required in order to oscillate the cantilever tip at kilohertz frequencies and nanometer-scale amplitudes, whilst simultaneously controlling the distance between the probe and the sample surface. Simply, the ‘cantilever tune’ mode of the Bioscope II AFM instrument can be used. The advantage of using AFM for indentation over nanoindentation is that a higher spatial resolution is obtained and AFM techniques are considered better suited to measuring the properties of extremely compliant materials (Akhtar et al., 2011).

The mean values for elastic modulus determined in this study lie within the range that has been reported in the literature for the aorta using indentation-based techniques but on full thickness sections of tissue (Hemmasizadeh et al., 2012, Matsumoto et al., 2004; McKee et al., 2011). McKee et al. reviewed and collated data from a number of studies on vascular tissues and reported an average value of approximately 125 kPa. None of these studies compare age-related changes in the aortic wall but provide suitable data for comparison with our work. The study by Matsumoto et al. focussed solely on characterising the MLU in porcine aorta (2–3 mm thick specimens) with a micro-indentation device reported a range of 50–180 kPa (Matsumoto et al., 2004), which also fits in within the range in our study. Hemmasizadeh et al. (2012) used a nanoindentation technique on full thickness specimens and reported data for the ‘outer’ (adventitia) and inner (‘media’) layers of the porcine thoracic aorta. Their elastic modulus measurements, corresponding to the medial layer, were around 60 kPa. Here, we demonstrate that we are able to obtain values within this range on thin cryosections of tissue (5 µm) hence confirming that the low force applied with FM-AFM does not lead to a significant substrate effect.

Both the *G*′and *G*″ followed the same trends as observed with the elastic modulus data. However, the trends in the data are dominated by the elastic properties of the aortic wall (*G*′). Both of these parameters increased with age, as has been reported with dynamic mechanical measurement techniques e.g. (Yin et al., 1983). With age *G*′ and *G*″ increased by the same relative amount as determined by the loss tangent data. Furthermore, the increase of *G*′and *G*″ with age was observed in both the lamellar and inter-lamellar regions of the vessel. Age-related arterial degradation is associated with fatigue failure of elastin and associated stiffening of the vessel (Greenwald, 2007) and a decline in viscoelastic properties (Yin et al., 1983). Our data suggests that the largest alterations to the viscoelastic properties of the aorta with age is largely localised to the inter-lamellar regions. This is not surprising given that the inter-lamellar region is an intricate meshwork of collagen fibres, elastin-fibres and smooth muscle cells, and changes in the interface between these components during ageing are thought to lead to changes in the viscoelasticity of the vessel wall (Silver et al., 2001). It is worth stressing that the measurements performed here establish the mechanical properties of elastin and collagen-rich hydrogel matrices, rather than the material properties of collagen or elastic fibres. Accordingly, we have previously reported that *in vitro*-generated collagen-based hydrogels or collagen-rich fibroblast-generated matrices display elastic modulus values much lower (Gavara et al., 2008, Petrie et al., 2012) than native collagen fibres (Gosline et al., 2002). Furthermore, as summarised by Holzapfel et al. (2002), numerous studies have reported that smooth muscle cells are primarily responsible for the viscoelastic behaviour of arteries.

The overall trends in the elastic properties that we found in the FM-AFM data are consistent with those we have reported previously using ultra-high frequency SAM (Graham et al., 2011), i.e. stiffening is largely localised to the inter-lamellar regions of the aortic wall. In the lamellar regions, 8% of the elastic modulus values were greater than 100 kPa in the young group as compared to 38% in the old group. In contrast, in the inter-lamellar regions, 8% of the elastic modulus values were greater than 100 kPa as compared to 50% in the old group. This large shift in the elastic modulus within the inter-lamellar regions fits with the histological observations that we reported previously (Graham et al., 2011), and well-established alterations that occur in the main structural components of the aorta with age, namely elastin and collagen (Greenwald, 2007). However, there are a number of differences between the two techniques which are summarised here. The FM-AFM method was conducted around 30 kHz whereas the SAM work was conducted around 1 GHz (Graham et al., 2011). The linear spatial resolution of SAM is determined by the operating frequency (around 1 μm) in our previous study. With FM-AFM, the spatial resolution is determined by the size of the probe. Although a 10 μm bead was used in this study with the FM-AFM setup, the estimated contact area is 1.6 μm^2^. As described by Gavara and Chadwick (2010), the tip radius and also the bead-sample gap determine the contact area. Thus, the effective areal spatial resolution is much lower than the 10 μm bead utilised in the study. SAM only provides a measure of the acoustic wave speed which is related to the elastic modulus of the tissue. The FM-AFM technique also provides a measure of the storage modulus and loss modulus, although at 30 kHz in the current setup. This is because the largest resonant peak displayed by the cantilever was used as the probing frequency. It is possible to conduct experiments at other frequencies by selecting cantilevers with a different shape or stiffness (Gavara and Chadwick, 2010). Crucially, compared with SAM, the FM-AFM was more sensitive to mechanical remodelling in the elastic lamellae. This may be related to the way in which the measurements have been carried out with SAM as with this approach images were, in some instances, difficult to interpret. Accordingly, only the central-third of pixels were analysed for the lamellar and inter-lamellar measurements. For the FM-AFM technique, accurate placement of the probe over the lamellar or inter-lamellar region was limited to the optical resolution of the associated epifluorescence microscope (Fig. 1). Finally, as stated earlier, SAM is a highly specialised technique whereas AFM is a much more accessible technique for many research groups. Hence, the FM-AFM method used in this study has an additional advantage in that it can be readily adopted using conventional AFM instruments. The FM-AFM method could therefore be used for a wide range of applications related to biological sample characterisation.

Future work on vascular tissue could be extended to determine the effects of ageing and disease on the arterial wall as a function of intraluminal pressure, given that changes in intraluminal pressure significantly affect the 3D microstructure of arterial walls (Walton et al., 2015).

## 5 Conclusions

FM-AFM is a useful tool for measure the mechanical properties of soft tissues such as the aorta and is a method that can be easily conducted using a conventional AFM by making use of ‘cantilever tuning’. We show that the technique can be used to probe age-related regional variations in the elastic and viscoelastic properties of ovine aorta. The FM-AFM data matches the trends in elastic behaviour we have previously reported with ultra-high frequency acoustic microscopy but provides new insight into viscoelastic changes in the aorta with age.

## Figures and Tables

**Table 1:** Mechanical properties determined for the young and old sheep aorta. Data is presented as geometric mean (geoSD).

	Young	Old	*P*-value
*E* (kPa)	42.9 (2.26)	113.9 (2.57)	*P*<0.0001
*G*′ (kPa)	14.3 (2.26)	38.0 (2.57)	*P*<0.0001
*G*″ (kPa)	14.5 (2.56)	32.8 (2.52)	*P*<0.0001

**Table 2:** Mean values for the mechanical properties determined for the lamellar and inter-lamellar regions of the medial for the young and old sheep aorta. Data is presented as geometric mean (geoSD).

	Lamellar (Young)	Lamellar (Old)	*P*-value (Lamellar)	Inter-lamellar (Young)	Inter-lamellar (Old)	*P*-value (Inter-lamellar)
*E* (kPa)	36.4 (2.22)	63.5 (2.95)	*P*<0.05	25.3 (3.39)	63.7 (2.76)	*P*<0.01
*G*′ (kPa)	12.1 (2.22)	24.3 (2.37)	*P*<0.05	8.42 (3.39)	21.2 (2.76)	*P*<0.01
*G*″ (kPa)	10.6 (2.77)	20.1 (2.67)	*P*<0.05	6.73 (3.98)	17.5 (3.02)	*P*<0.01
